# Management of iatrogenic esophageal perforations: a systematic review of non-surgical causes

**DOI:** 10.1007/s00464-026-12834-1

**Published:** 2026-04-27

**Authors:** Dimitrios Papaconstantinou, Sofia Theofilopoulou, Christiana Ioannou, Evgenia Mela, Stavros P. Papadakos, Georgios Tribonias, Tania Triantafyllou, Ioannis Rouvelas, Dimitrios Schizas

**Affiliations:** 1 Third Department of Surgery, National and Kapodistrian University of Athens, Attikon University Hospital, Athens, Greece; 2 First Department of Surgery, National and Kapodistrian University of Athens, Laikon General Hospital, Athens, Greece; 3 First Department of Propaedeutic Surgery, National and Kapodistrian University of Athens, Hippocration General Hospital, Athens, Greece; 4 First Department of Gastroenterology, National and Kapodistrian University of Athens, Laikon General Hospital, Athens, Greece; 5 Department of Gastroenterology, Red Cross Hospital, Athens, Greece; 6 Division of Surgery and Oncology, Department of Clinical Science, Intervention and Technology (CLINTEC), Karolinska Institutet, Solna, Sweden; 7 Department of Upper Abdominal Diseases, Karolinska University Hospital, Huddinge, Stockholm, Sweden

**Keywords:** Iatrogenic esophageal perforation, Endoscopy-related complications, Esophageal stenting, Systematic review

## Abstract

**Background:**

Iatrogenic esophageal perforations (IEP) are uncommon but potentially fatal complications, most frequently related to endoscopic procedures. Previous systematic reviews have evaluated esophageal perforations as a heterogeneous entity, frequently combining spontaneous, traumatic, surgical, and iatrogenic causes. However, outcome estimates specific to non-surgical, iatrogenic perforations remain less clearly defined. This study aimed to systematically review and meta-analyze the causes, management strategies, and outcomes of patients managed for non-surgical IEPs.

**Methods:**

A systematic search of MEDLINE, Embase, CENTRAL, Scopus, and ClinicalTrials.gov was performed from inception to September 2025 in accordance with PRISMA guidelines. Studies reporting clinical outcomes of adult patients with iatrogenic, non-surgical esophageal perforations were included. Postoperative surgical perforations and case reports were excluded. Primary outcomes were procedure type leading to perforation, overall mortality, and mortality according to treatment strategy. Random-effects meta-analyses were conducted for pooled estimates.

**Results:**

Twenty-five studies encompassing 596 patients were included. Interventional procedures accounted for 71.1% of perforations, while diagnostic procedures accounted for 28.9%. Diagnostic endoscopy was significantly less likely to cause perforation compared with interventional procedures (OR 0.20, 95% CI 0.08–0.45). The pooled overall mortality rate was 9.75% (95% CI 5.9%–13.6%). Among studies reporting treatment-specific outcomes, endoscopic-based management was employed in 48.1% of patients, most commonly involving esophageal stenting, and was associated with a pooled mortality of 11.37% (95% CI 2.84%–19.9%). Surgical management was used in 51.9% of cases, predominantly primary repair or esophagectomy, with a pooled mortality of 11.58% (95% CI 7.13%–16.03%).

**Conclusions:**

Non-surgical IEP occurs predominantly after interventional procedures and remains associated with substantial mortality approaching 10%. Endoscopy-based management, particularly with stent placement, has become central to treatment and demonstrates outcomes comparable to surgery in selected patients, while surgery remains as a salvage option. Management should be individualized, and further high-quality studies are needed to refine treatment algorithms.

**Supplementary Information:**

The online version contains supplementary material available at 10.1007/s00464-026-12834-1.

Esophageal perforation is an uncommon but life-threatening event, with an estimated incidence of 1–5 cases per 100,000 individuals annually [1]. Among its various causes, iatrogenic esophageal perforations (IEP) predominate, accounting for up to 50% of cases depending on the patient population studied [2, 3]. This is largely due to the widespread adoption of upper gastrointestinal endoscopic procedures, which now encompass both diagnostic and therapeutic interventions. Although the risk of perforation during diagnostic endoscopy with flexible instruments remains low, the incidence increases with procedural complexity, particularly when interventions such as pneumatic dilation, stent placement, endoscopic mucosal or submucosal resection, or ablative therapies are performed. The risk is further increased in patients with underlying esophageal disease, such as malignancy, where the structural integrity of the organ is already compromised [1, 2].

Despite advances in diagnostic modalities, therapeutic techniques, and intensive care, esophageal perforation continues to be associated with substantial morbidity and mortality [1, 4], often as a result of septic complications [5]. In cases of IEP, such adverse outcomes can impose significant patient burden and carry medicolegal implications, especially when the underlying pathology is benign. Prompt diagnosis and coordinated multidisciplinary management are therefore essential.

Currently, management strategies vary widely, ranging from endoscopic stent placement and clip closure to surgical resection [6]. The choice of treatment depends on factors such as the underlying pathology, timing of diagnosis, and the patient’s age and physiological status, but ultimately rests on local expertise, as no standardized management algorithm exists. Moreover, the literature on IEP is limited and heterogeneous due to the rarity of the condition, with key disease parameters remaining poorly defined.

The aim of this systematic review was to synthesize evidence on endoscopy-related causes of iatrogenic perforation to better characterize associated clinical parameters, including the type of modality leading to perforation, overall mortality rates, and the efficacy of treatment strategies employed.

## Materials and methods

### Search strategy

A systematic literature search of the MEDLINE, Embase, CENTRAL and Scopus databases, as well as the Clinicaltrials.gov register was conducted from inception to September 2025, using the terms “esophagus[Mesh],” “Iatrogenic,” “Esophageal Perforation”[Mesh], “Wounds and Injuries”[Mesh], and “trauma,” combined using the Boolean operators AND/OR as appropriate for each database. Following duplicate removal, the abstracts were independently screened by two authors (ST, CI) for potentially eligible studies, while a third author (DP) ensured completeness and minimized the risk of missing relevant studies. The reference lists of studies within the explored topic were further manually checked for additional articles of relevance.

These systematic review and meta-analysis were conducted in accordance with PRISMA guidelines [7] and were prospectively registered in the International Prospective Register of Systematic Reviews in 2024 (PROSPERO ID: CRD420251143434).

### Eligibility and exclusion criteria

The present systematic review and meta-analysis included studies reporting clinical outcomes in patients with iatrogenic esophageal perforations, particularly those resulting from intraluminal instrumentation of the esophagus during diagnostic or interventional procedures. Perforations occurring as postoperative surgical complications were considered beyond the scope of the present review. However, to maximize inclusivity, studies were included if a small proportion of surgical cases could not be disaggregated from a broader relevant cohort, provided that these cases comprised less than one-fifth of the total study population.

The predetermined list of exclusion criteria that was utilized to guide the study selection process was as follows: (1) non-clinical studies and case reports, (2) studies on pediatric patient populations, (3) studies focusing exclusively on postoperative iatrogenic perforations of the esophagus, (4) studies from the same institution with patient overlap, (5) studies in which iatrogenic causes of perforation were intermixed with non-iatrogenic etiologies and separation of data was not feasible, and (6) studies not reporting data on any outcomes of interest.

### Outcomes of interest and data collection

Primary outcomes of interest were the type of procedure leading to the perforation (diagnostic or interventional), global 90-day or in-hospital mortality rates, and the type of treatment pursued along with its associated mortality. Secondary outcomes of interest were patient demographics, the location of the perforation within the esophagus, and the specific treatment modalities utilized.

Studies deemed potentially eligible by the abstract screening process were extracted and evaluated in full text by two authors (ST, CI) for inclusion in the data synthesis. A third author (DP) acted as a referee in cases of disagreement and ensured data extraction accuracy. After the final study selection was complete, all data pertaining to the outcomes of interest were entered into standardized Excel spreadsheets (Microsoft, Redmond, Washington, USA) for data tabulation.

### Risk of bias assessment

Assessment of the risk of bias of included studies was qualitatively performed using the ROBINS-E tool developed by the Cochrane Collaboration [8]. This tool applies to non-randomized studies of exposure and evaluates the individual risk of bias for each included study across seven domains: (1) bias due to confounding, (2) bias arising from measurement of the exposure, (3) bias in the selection of patients into the study, (4) bias due to post-exposure interventions, (5) bias due to missing data, (6) bias in measurement of the outcome, and (7) bias in selection of the report result. Two authors (DP, DS) undertook the risk of bias assessment, sequentially evaluating each study for each of the ROBINS-E domains, allocating a grade that could be either low, moderate or serious risk of bias. All cases of disagreement were resolved by reaching a common consensus.

### Statistical analysis

Descriptive statistics, in the form of absolute numbers and percentages, were used to present the outcomes of interest. Data synthesis was performed using a random-effects meta-analysis (DerSimonian and Laird) due to expected imbalances in patient baseline factors and interstudy heterogeneity. Dichotomous outcomes (i.e., interventional versus diagnostic causes of perforation) were analyzed by standard meta-analysis which computes pooled odds ratios (OR) and corresponding 95% confidence intervals (CI). For pooled analyses of mortality rates, a meta-analysis of proportions was implemented, with a continuity correction applied to account for zero-event studies.

Interstudy statistical heterogeneity was assessed using Higgins’ *I*^2^ statistic, with values below 50% indicating moderate heterogeneity and values at or above 50% indicating significant heterogeneity. Funnel plots were constructed for all analyses and were visually inspected for asymmetries. Publication bias was evaluated using the Doi plot and quantified via the Luis Furuya-Kanamori (LFK) index [9], where a value beyond ± 2 indicates major asymmetry; Egger’s test was also performed for supplementary assessment. All statistical analyses were performed using Stata v17 (StataCorp. 2021. Stata Statistical Software: Release 17. College Station, TX: StataCorp LLC). A p-value of 0.05 or less was considered statistically significant.

## Results

**Fig. 1:**
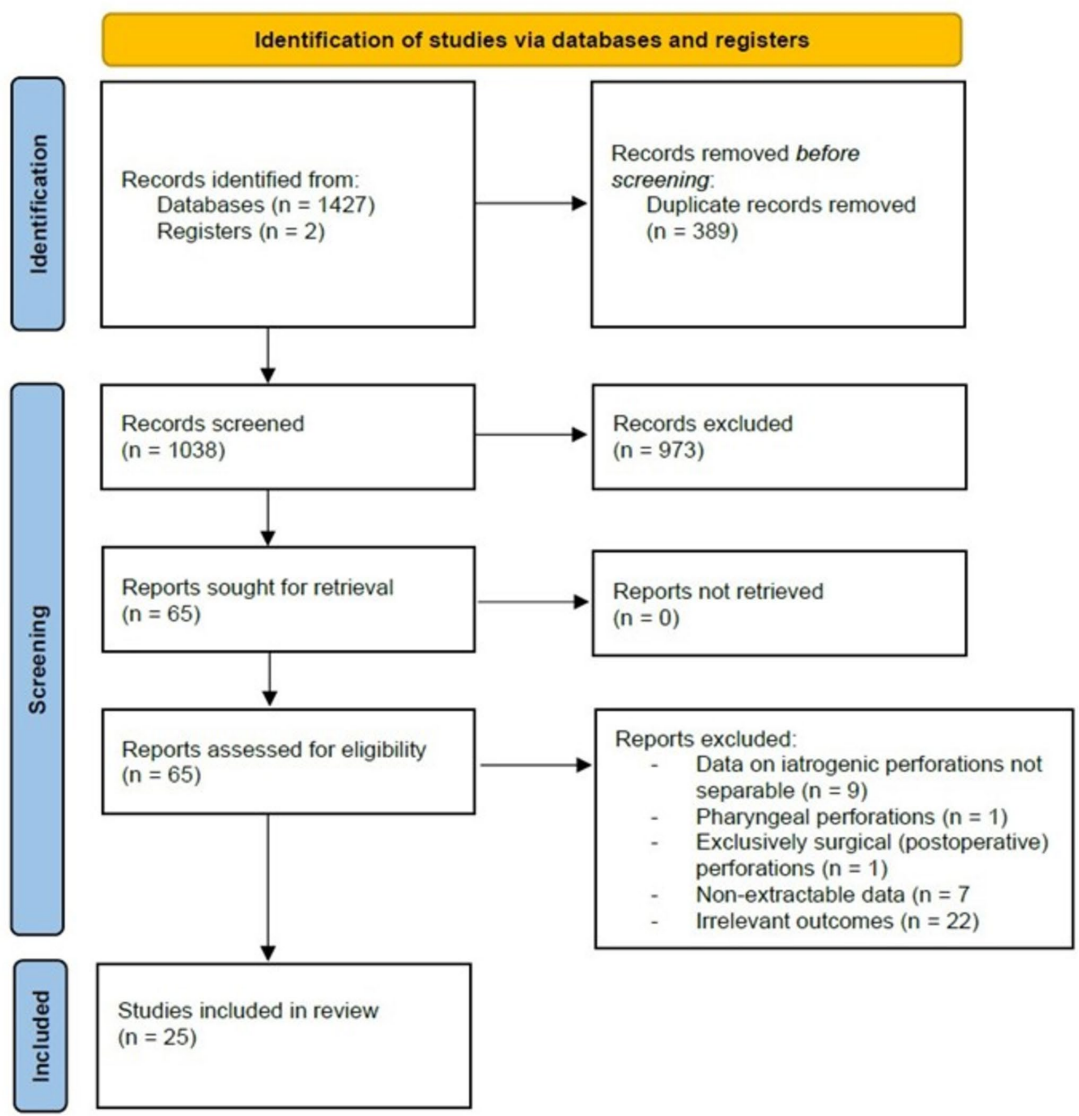
PRISMA flowchart of study selection

After screening 1038 unique abstracts and completing the study selection process, 25 studies [2, 10–33] totaling 596 patients were deemed eligible for inclusion in the final analysis (Fig. 1). Study publication dates ranged from 1999 to 2024, with sixteen studies originating from Europe, six from North America, and three from Asia. Median reported age ranged from 49 to 69 years of age, with the cohort exhibiting a slight male predominance (52.7% males, 47.3% females). Patient characteristics are summarized in Table 1.

**Table 1:** Baseline patient demographics and perforation characteristics

Author	Country	Number of patients (*n*)	Age (years)	Gender (M/F)	Procedures leading to perforation *n*, (%)		Perforation location *n*, (%)			Mortality
					Diagnostic	Interventional	Proximal	Middle	Distal	
Karstens et al., [11]	Germany	39	69 (24–87)	12/27	23 (58.9)	16 (41.1)	8 (20.5)	15 (38.5)	16 (41)	8 (20.5)
Ko et al., [10]	USA	2	52 (44–60)	1/1	2 (100)	0	N/A	N/A	N/A	0
Levin et al., [12]	Italy	24	69 (29–89)	15/9	6 (25)	18 (75)	24 (100)	0	0	3 (12.5)
Lindeman et al., [13]	Austria	70	N/A	N/A	30 (42.9)	40 (57.1)	8 (11.4)	22 (31.4)	40 (57.1)	N/A
Montminy et al., [33]	USA	32	61	15/17	N/A	N/A	8 (25)	6 (18.8)	18 (56.3)	4 (12.5)
Nijhuis et al., [14]	Netherlands	4	61	0/4	0	4 (100)	0	0	4 (100)	0
Okten et al., [15]	Turkey	25	N/A	N/A	7 (28)	18 (72)	8 (32)	17 (68)	0	6 (24)
Siersema et al., [17]	Netherlands	3	43 (38–68)	1/2	2 (66.7)	1 (33.3)	0	0	3 (100)	0
Tomaselli et al., [18]	Austria	38	N/A	N/A	17 (44.7)	21 (55.3)	0	36 (94.7)	2 (5.3)	6 (15.8)
Vallböhmer et al., [19]	Germany	28	N/A	N/A	11 (39.3)	17 (60.7)	3 (10.7)	15 (53.6)	10 (35.7)	2
Vidarsdottir et al., [2]	Iceland	15	N/A	N/A	4 (26.7)	11 (73.3)	N/A	N/A	N/A	N/A
White et al., [20]	USA	10	60 (40–86)	N/A	0	10 (100)	0	7 (70)	3 (30)	0
Zenga et al., [21]	USA	19	N/A	N/A	7 (36.8)	12 (63.2)	19 (100)	0	0	0
Aghajanzadeh et al., [23]	India	8	56.5 (28 – 63)	4/4	4 (50)	4 (50)	N/A	N/A	N/A	0
Ayed et al., [24]	Kuwait	10	38 (29 – 53)	N/A	6 (60)	4 (40)	0	0	10 (100)	1 (10)
Bresadola et al., [32]	Italy	7	59 (53 – 82)	3/4	N/A	N/A	4	3	0	0
Fernandez et al., [25]	Germany	75	64.4 (2–90)	44/31	2 (3)	73 (97)	7 (9.5)	25 (33.3)	43 (57.2)	14 (18.7)
Fischer et al., [26]	Germany	6	56.5 (25 – 81)	3/3	0	6 (100)	0	2 (33.3)	4 (66.7)	N/A
Freeman et al., [27]	USA	8	51 (31 – 91)	N/A	3 (37.5)	5 (62.5)	N/A	N/A	N/A	0
Freeman et al., [28]	USA	60	60	37/23	0	60 (100)	0	60 (100)	0	3 (5)
Hasan et al., [29]	UK	26	59 (16 – 92)	12/14	1 (4)	25 (96)	2 (7.7)	17 (65.4)	7 (26.9)	4 (15.4)
Hauge et al., [30]	Norway	21	66 (26 – 89)	12/9	4 (19)	17 (81)	N/A	N/A	N/A	1 (4.8)
Heits et al., [16]	Germany	4	68 (61 – 75)	N/A	N/A	N/A	2 (50)	2 (50)	0	1 (25)
Huber-lang et al., [22]	Germany	8	63.5 (45 – 68)	N/A	7 (87.5)	1 (12.5)	2 (25)	5 (62.5)	1 (12.5)	2 (25)
Jougon et al., [31]	France	54	62	33/21	24 (44.4)	30 (55.6)	13 (30.2)	8 (18.6)	22 (51.2)	14 (26)

Age is presented as median (range)

Risk of bias was assessed for the entirety of the included studies using the ROBINS-E tool (Supplementary Figure S1). Most studies were retrospective single-center case series, with “some concerns” related to confounding and the potential for selection of participants. A smaller subset of studies, particularly small case series and those with highly selected patient cohorts, were judged to be at high risk of bias. Overall, risk of bias across all studies was moderate to serious, reflecting the inherent limitations of the observational design and the small sample size of the included studies.

### Causes and characteristics of the perforations

**Fig. 2:**
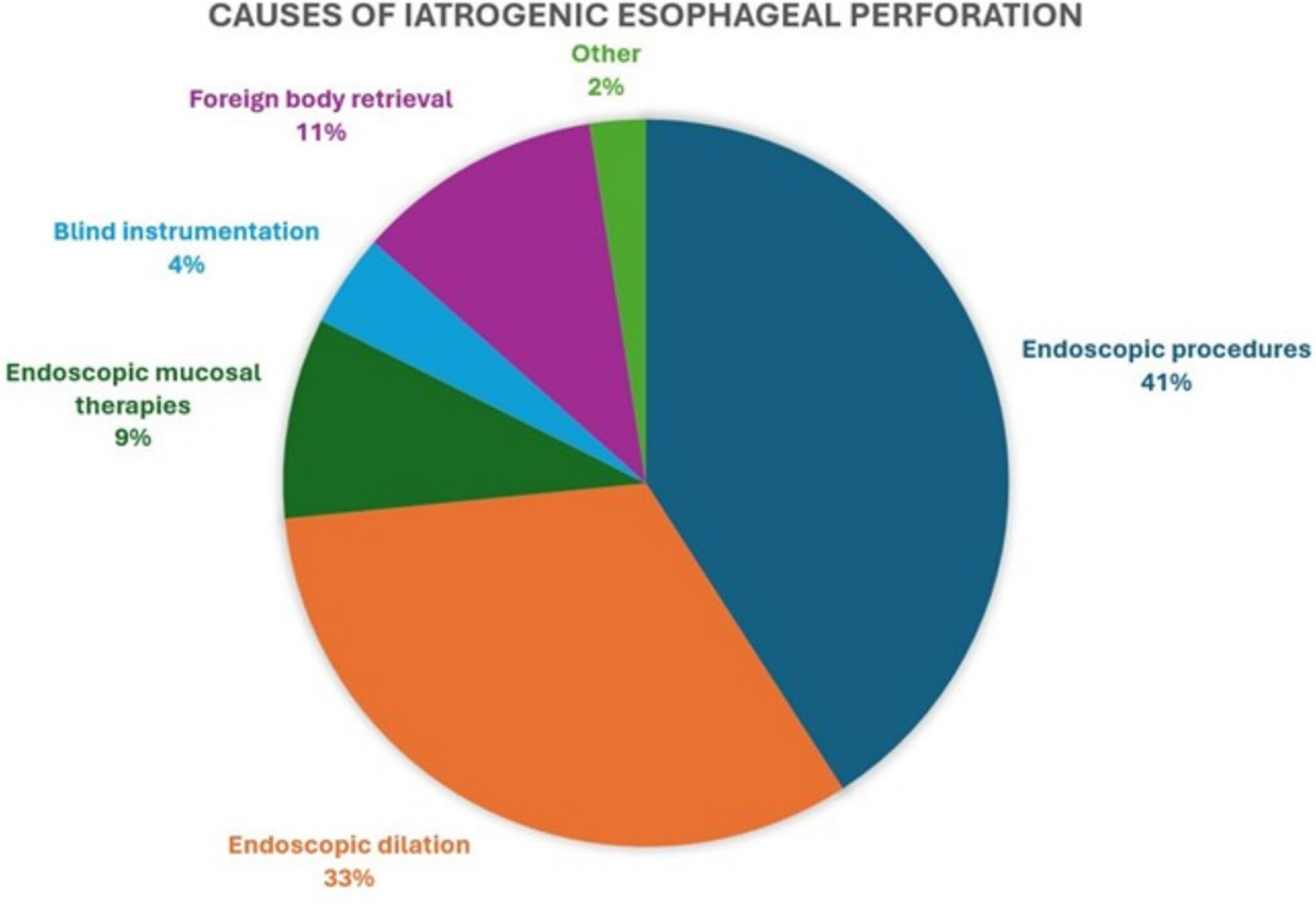
Types of procedures leading to iatrogenic esophageal perforations

**Fig. 3:**
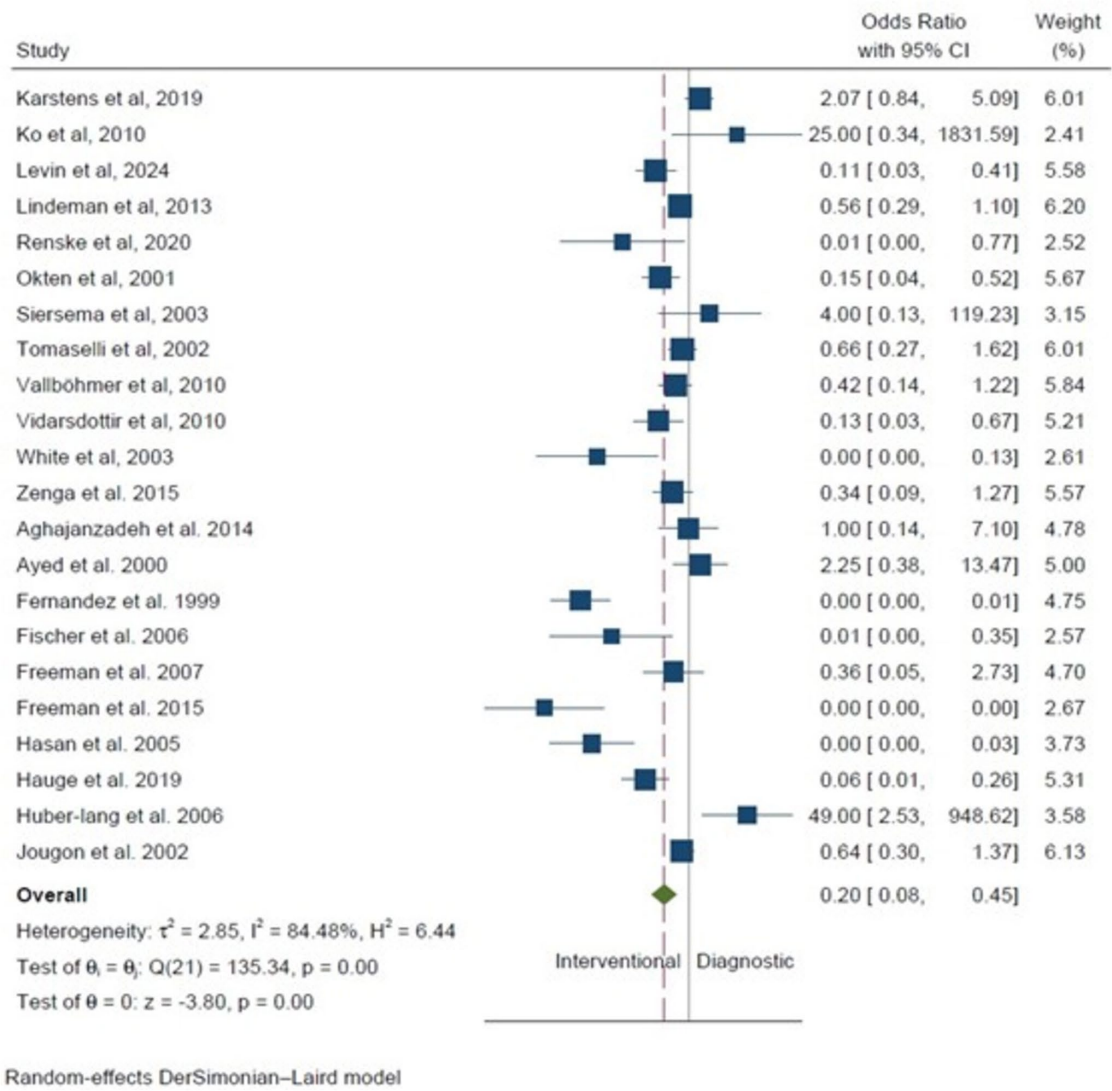
Forest plot of interventional versus diagnostic procedures as the cause of the esophageal perforation

Twenty-two studies [2, 10–15, 17–31, 33] reported on the type of procedure preceding the perforation, with interventional procedures being predominant (*n* = 393, 71.1%), compared with diagnostic procedures (*n* = 160, 28.9%). Regarding the specific type of procedure leading to the perforation, the majority (41%) were endoscopic procedures including simple endoscopy, endoscopic ultrasonography of the upper gastrointestinal tract, and endoscopic retrograde cholangiopancreatography with or without stent placement or removal (Fig. 2). The second most common cause was endoscopic dilation (33%), followed by endoscopic mucosal therapies (such as endoscopic mucosal resection, variceal sclerotherapy, or photodynamic therapy), endoscopic foreign body retrieval attempts (11%), and blind esophageal instrumentation (such as esophageal intubation, nasogastric tube passage, or aggressive bougienage). Meta-analysis of the studies reporting procedure type as the cause of the esophageal perforation revealed that diagnostic procedures were 80% less likely to be the underlying cause of injury (OR 0.2, 95% CI 0.08–0.45, *p* < 0.001, Fig. 3). Significant interstudy heterogeneity was encountered with *I*^2^ = 84.5%.

**Fig. 4:**
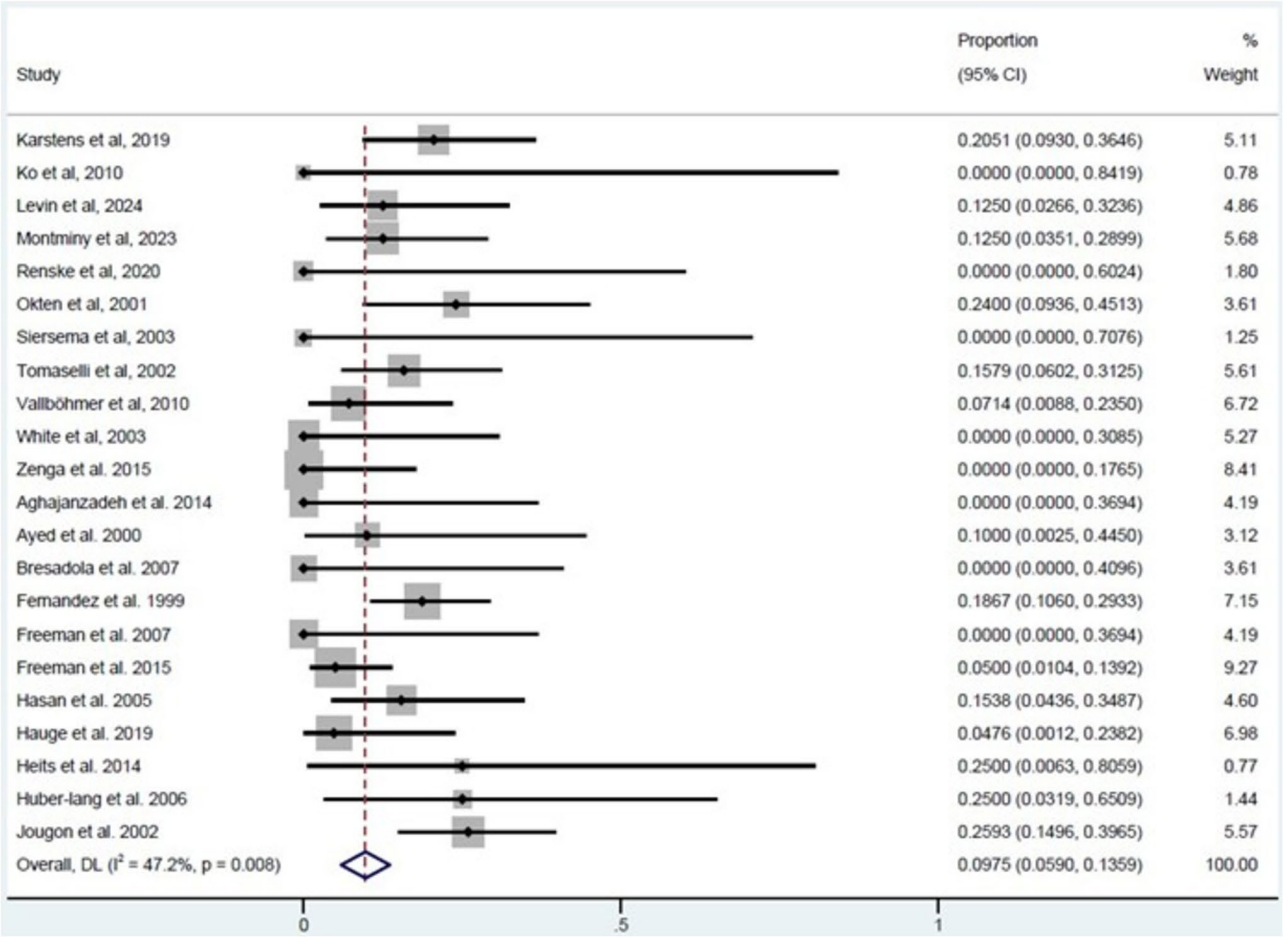
Forest plot of the pooled mortality rate for the entire patient population

A total of 69 fatalities were reported among twenty-two studies, with mortality rates ranging from 0 to 25% (Table 1). Pooled global mortality rates were 9.75% (95% CI 5.9 to 13.59, Fig. 4) with moderate interstudy heterogeneity (*I*^2^ = 47.2%).

### Treatment approaches and outcomes

A subset of eighteen studies reported on the type of treatment strategy employed. Surgical management was pursued slightly more often (*n* = 224, 51.9%) than endoscopy-based management (*n* = 208, 48.1%). Among patients managed conservatively, cessation of oral intake, administration of antibiotics, and liberal use of drains were commonplace. Additionally, stent usage was reported in 88 patients (91.2%), while endoscopic vacuum-assisted or clip closure of the perforations were sporadically reported (*n *= 3 and *n* = 5, respectively). The pooled mortality rate among patients undergoing endoscopic management was 11.37% (95% CI 2.84% to 19.9%, *I*^2^ = 70%).

In surgically treated patients, primary suturing of the defect was the procedure of choice in most cases (*n* = 81, 62.8%), followed by esophagectomy with or without immediate reconstruction (*n* = 33, 25.6%). Surgical exploration for debridement and drainage was employed in 14 cases (10.6%), while a single case was managed by omental patching (Table 2). The pooled mortality rate for surgically treated patients was 11.58% (95% CI 7.13% to 16.03%, *I*^2^ = 0).

**Table 2:** Detailed treatment approaches and mortality outcomes

Study	Conservative treatment *n*, (%)	Types of endoscopic modalities n, (%)			Mortality *n*, (%)	Surgical treatment *n*, (%)	Types of surgical modalities *n*, (%)			Mortality *n*, (%)
		Stent	EndoVac	Other			Primary repair	Resection	Other	
Ko et al., [10]	N/A	N/A	N/A	N/A	N/A	2 (100)	2 (100)	N/A	N/A	0
Levin et al., [12]	9 (37.5)	N/A	N/A	N/A	1 (4.2)	15 (62.5)	6 (40)	N/A	Exploration; 9 (60)	2 (13.3)
Montminy et al., [33]	28 (87.5)	17 (60.7)	1 (3.1)	Endoclip; 3 (10.7)	N/A	4 (12.5)	4 (100)	N/A	N/A	N/A
Nijhuis et al., [14]	4 (100)	N/A	2 (50)	Endoclip; 2 (50)	0	N/A	N/A	N/A	N/A	N/A
Siersema et al., [17]	3 (100)	3 (100)	N/A	N/A	0	N/A	N/A	N/A	N/A	N/A
Tomaselli et al., [18]	3 (7.9)	3 (100)	N/A	N/A	1 (33.3)	35 (92.1)	21(60)	14 (40)	N/A	5 (14.3)
Vallböhmer et al., [19]	14 (50)	8 (57)	N/A	N/A	1 (7.1)	14 (50)	7 (50)	7 (50)	N/A	1 (7.1)
White et al., [20]	9 (90)	9 (100)	N/A	N/A	0	1 (10)	N/A	N/A	Omental patch; 1 (100)	1 (100)
Aghajanzadeh et al., [23]	5 (62.5)	1 (20)	N/A	N/A	0	3 (37.5)	3 (100)	N/A	N/A	0
Ayed et al., [24]	N/A	N/A	N/A	N/A	N/A	10 (100)	10 (100)	N/A	N/A	1 (10)
Bresadola et al., [32]	2 (28.6)	N/A	N/A	N/A	0	5 (71.4)	2 (40)	3 (60)	N/A	0
Fernandez et al., [25]	50 (66.7)	N/A	N/A	N/A	8 (16)	25 (33.3)	21 (84)	4 (16)	N/A	6 (24)
Fischer et al., [26]	6 (100)	6 (100)	N/A	N/A	N/A	N/A	N/A	N/A	N/A	N/A
Freeman et al., [28]	30 (50)	30 (100)	N/A	N/A	1 (3.3)	30 (50)	N/A	N/A	N/A	2 (6.7)
Hasan et al., [29]	26 (100)	N/A	N/A	N/A	4 (15.4)	N/A	N/A	N/A	N/A	N/A
Hauge et al., [30]	15 (71.4)	11 (73.3)	N/A	N/A	1 (6.7)	6 (28.6)	2 (33.3)	2 (33.3)	Debridement; 2 (33.3)	0
Huber-lang et al., [22]	N/A	N/A	N/A	N/A	N/A	8 (100)	2 (25)	3 (37.5)	Debridement; 3 (37.5)	2 (25)
Jougon et al., [31]	3 (5.6)	N/A	N/A	N/A	3 (100)	51 (94.4)	N/A	N/A	N/A	11 (21.6)

### Publication bias

In the evaluation for publication bias for the comparison between interventional and diagnostic procedures leading to the IEP, visual inspection of the funnel plot revealed some asymmetry (Supplementary Figure S2). The Doi plot with an LFK index of –2.0 indicated major asymmetry (Supplementary Figure S3), suggestive of publication bias. Egger’s regression test yielded a *p*-value of 0.12, not supporting the presence of small-study effects. To further explore potential small-study effects, we applied Duval and Tweedie’s trim-and-fill method [34], which did not materially alter the pooled estimates. Overall, the results suggest that publication bias, if present, is unlikely to have substantially influenced the findings. No major asymmetries were observed in any of the other evaluated outcomes (pooled mortality, endoscopic management mortality, and surgical management mortality).

## Discussion

In this systematic review focusing on non-surgical causes of IEP, the expansion of interventional endoscopy has been identified as the main driver behind the increasing incidence of these injuries over the past decade [1, 4, 35]. This rise in incidence likely reflects the wider use of minimally invasive modalities for conditions such as malignancy or benign esophageal stenosis, which would otherwise require open surgical treatment. The development of IEP is associated with considerable morbidity and a risk of death, which, based on the pooled results of the present review, may be as high as 10%. While several reviews have summarized the management of esophageal perforation in general, few have focused specifically on iatrogenic, non-surgical causes, which are a subgroup of increasing clinical relevance due to the expansion of therapeutic endoscopy. However, these reviews have often combined multiple etiologies, potentially introducing clinical heterogeneity. To our knowledge, this is the first systematic review to synthesize outcomes of iatrogenic esophageal perforations exclusively, thereby reducing etiologic heterogeneity and providing more homogeneous pooled outcome estimates within this specific clinical context.

Interestingly, mortality rates have shown a downward trend, likely driven by the same factors underlying the increased incidence: the expansion of interventional endoscopy. In particular, the advent of esophageal stents has broadened the scope of conservative management, even in cases of overt perforation. The propensity score-matched study by Freeman et al. [27] highlighted the potential advantages of stenting, including shorter hospitalization, earlier oral intake, and substantially reduced complication rates compared with surgery. Stent placement has become so prevalent that it now represents the cornerstone of conservative management. In our pooled sample, 91% of endoscopically treated patients underwent stent placement alongside supportive measures, with especially low mortality reported in studies where stenting was the exclusive modality, particularly in more recent publications where operator experience and device quality have improved (Table 2). The development of fully covered self-expandable metal stents (FCSEMS) along with clip fixation to bridge the esophageal defect has been found to carry favorable safety profiles [36]. Other endoscopic techniques, such as endoscopic vacuum therapy and clip placement, or even endoscopic suturing devices [37] further supplement the available toolkit for managing IEP. When a perforation is recognized during the endoscopic procedure, immediate endoscopic closure is recommended as the standard of care, and techniques such as the through-the-scope or over-the-scope clips may be useful for smaller, well-demarcated defects [38]. However, their role in IEP remains secondary compared with stents [14, 33].

Endoscopic vacuum therapy is another modality that has gained attention in the management of esophageal perforations of both endoscopic and surgical origin. First applied [39] in the management of rectal luminal defects, expansion of its application in the upper gastrointestinal tract has shown promising results in small patient series, with healing of the perforation being achievable within an acceptable timeframe of around 14–16 days [40, 41]. However, evidence on the efficacy of endoscopic vacuum therapy remains limited, and its representation in the studies included in this review was sparse. Consequently, the present pooled estimates predominantly reflect stent-based management strategies. Although comparative analyses have suggested favorable outcomes with EndoVAC in selected settings [42, 43], these data remain heterogeneous and were not sufficiently reported within our included cohorts to permit meaningful evaluation. Endoscopic vacuum therapy may therefore represent an adjunctive or step-up option in specialized centers, but its role relative to stenting requires further clarification in prospective studies.

Although pooled mortality rates appeared similar between surgical and endoscopic management, direct comparison is limited by case selection bias since surgery is usually reserved for larger perforations and for patients with sepsis or failed endoscopic control. This observation underscores that endoscopic therapy is not universally applicable. This is especially relevant for perforations located in regions less amenable to stenting, such as the cervical esophagus and hypopharynx. In such settings, surgery may be more appropriate, particularly as many patients present with sepsis that precludes conservative measures [12, 21]. Surgery is also more commonly applicable in cases of early diagnosis, but it remains an important salvage option when conservative therapy fails, within a multimodal treatment framework [44].

Substantial statistical heterogeneity was observed in certain pooled analyses, particularly in the comparison between interventional and diagnostic procedures and in mortality among endoscopically managed patients. This variability likely reflects differences in patient selection, perforation severity, timing of diagnosis, and institutional management strategies across studies. Given the retrospective nature of the available data and the absence of standardized treatment algorithms, such heterogeneity is expected. Although random-effects models were applied to account for between-study variation, the pooled estimates should be interpreted as representing average effects across diverse clinical settings rather than uniform outcomes.

Among surgical strategies, primary repair emerged as the most frequently employed, sometimes reinforced with an omental patch [20]. This approach is rational, given that many IEPs are recognized early and often involve small defects. However, surgical treatment should be individualized. Resectional strategies remain appropriate in the presence of malignancy or non-viable tissue. However, source control and patient stabilization should be prioritized over immediate reconstruction in cases of delayed diagnosis with established sepsis, given the high associated mortality [44].

Cervical perforations, although generally less lethal, are more often treated surgically, as this avoids thoracotomy and is therefore less likely to increase morbidity. In contrast, thoracic perforations carry a higher operative threshold since many are amenable to endoscopic stenting, while surgical access and perioperative stabilization are particularly demanding in critically ill patients. Adequate drainage remains pivotal across strategies, and multiple endoscopic reinterventions may also be required to ensure adequate stent coverage and source control. Ultimately, treatment decisions should be individualized according to the timing of diagnosis, perforation site, patient condition, comorbidities, and the availability of endoscopic and surgical expertise; the latter being crucial for achieving low mortality.

This review has several limitations that need to be acknowledged. Data were limited, with most included studies being retrospective, single-center cohorts with small sample sizes. As such, unmeasured confounding is likely, given the clinical heterogeneity of IEP. For example, the impact of center experience and time of diagnosis was not presently evaluated. There was also wide variation in management strategies, even within single studies, and observable shifts over time, with more recent cohorts emphasizing endoscopic treatment. Contemporary endoscopic techniques such as endoscopic vacuum therapy, as well as combined or step-up approaches integrating endoscopic and surgical modalities, were sparsely and inconsistently reported in the included studies, precluding meaningful evaluation of these strategies within the present analysis. Inconsistent definitions of IEP were another issue: some series included surgery-related injuries, while others focused strictly on complications of diagnostic or therapeutic endoscopy. Although methodological efforts were made to minimize this variation, the risk of patient selection bias remains. It should also be emphasized that although pooled mortality rates for the conservative/endoscopic and surgical treatment groups were numerically equivalent, the underlying imbalances in patient selection do not permit a direct comparison and thus no inference regarding equivalence or superiority of management approaches can be drawn from the present analysis. Furthermore, substantial heterogeneity in some pooled analyses limits the precision of the summary estimates and reflects underlying clinical variability that could not be fully explored within the constraints of the available data. In addition, long-term outcomes such as dysphagia, stricture formation, and quality of life were underreported, limiting evaluation of functional recovery. Finally, ROBINS-E assessment indicated moderate to serious risk of bias in most studies, and the possibility of publication bias cannot be excluded.

In conclusion, endoscopy-related IEP is a complication predominantly associated with interventional procedures, which are associated with a substantially increased risk compared with diagnostic endoscopy. This risk should therefore be explicitly communicated to patients undergoing such procedures. Mortality remains considerable and may reach 10%, making prompt diagnosis and appropriate management essential. Treatment should be individualized, taking into account patient factors, perforation location, and comorbidities. Endoscopic management with stenting appears to reduce mortality and has become central to therapy, yet surgery remains indispensable, particularly as salvage. Given the rising incidence of IEP with the expansion of therapeutic endoscopy, further clinical reporting with clear definitions and standardized outcome measures is needed to refine management algorithms and improve patient care.

## Supplementary Information

Below is the link to the electronic supplementary material.

Supplementary file1 (DOCX 622 KB)
