# Supplementary material for: Management of iatrogenic esophageal perforations: a systematic review of non-surgical causes
Source: Surg Endosc. 2026 Apr 27;40(5):3709–20. doi: 10.1007/s00464-026-12834-1 (PMC13161298; doi:10.1007/s00464-026-12834-1)
Supplement: Supplementary file 1 — Supplementary file1 (DOCX 622 KB) [file 464_2026_12834_MOESM1_ESM.docx]

**
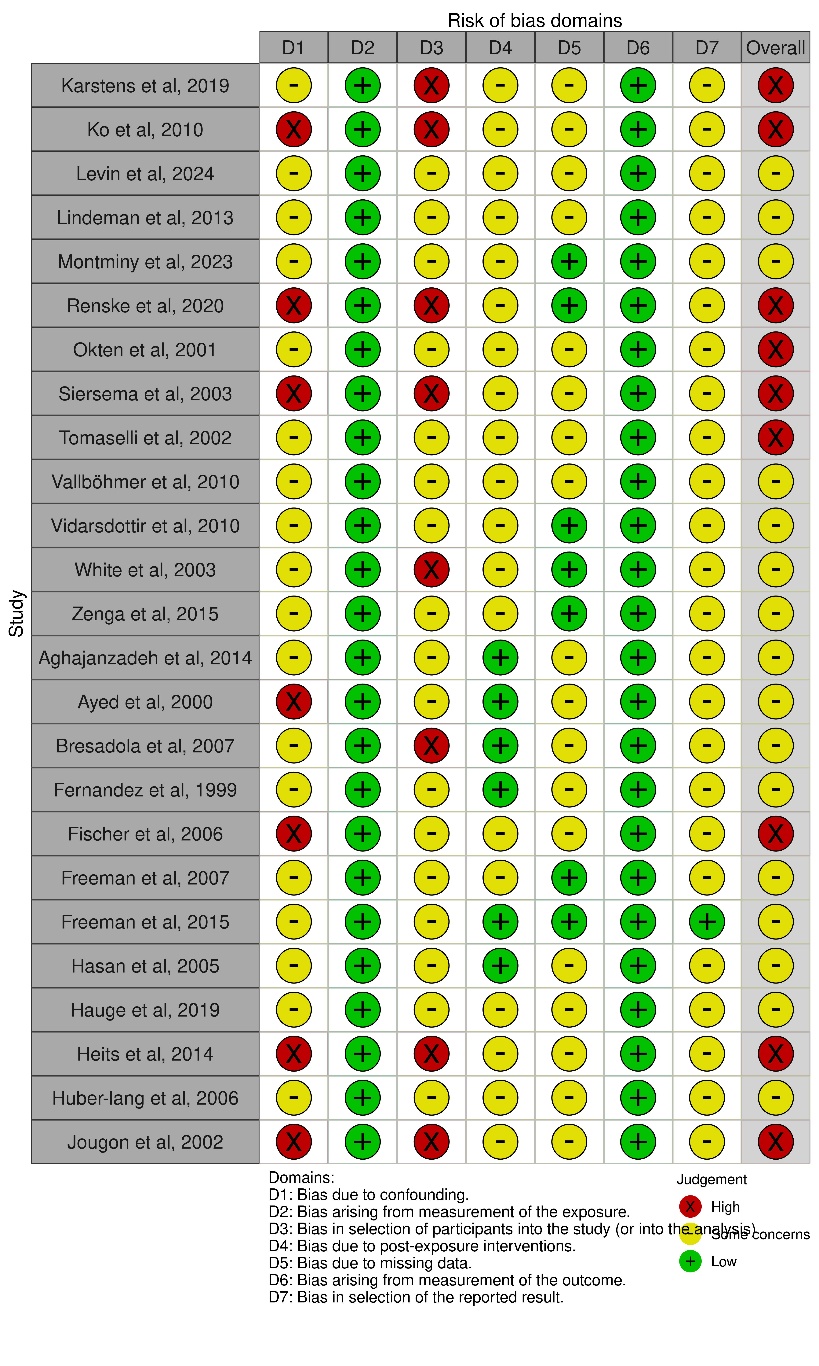
**

**Supplementary Figure S1**. Risk of bias assessment of studies of exposure effects using the ROBINS-E tool.


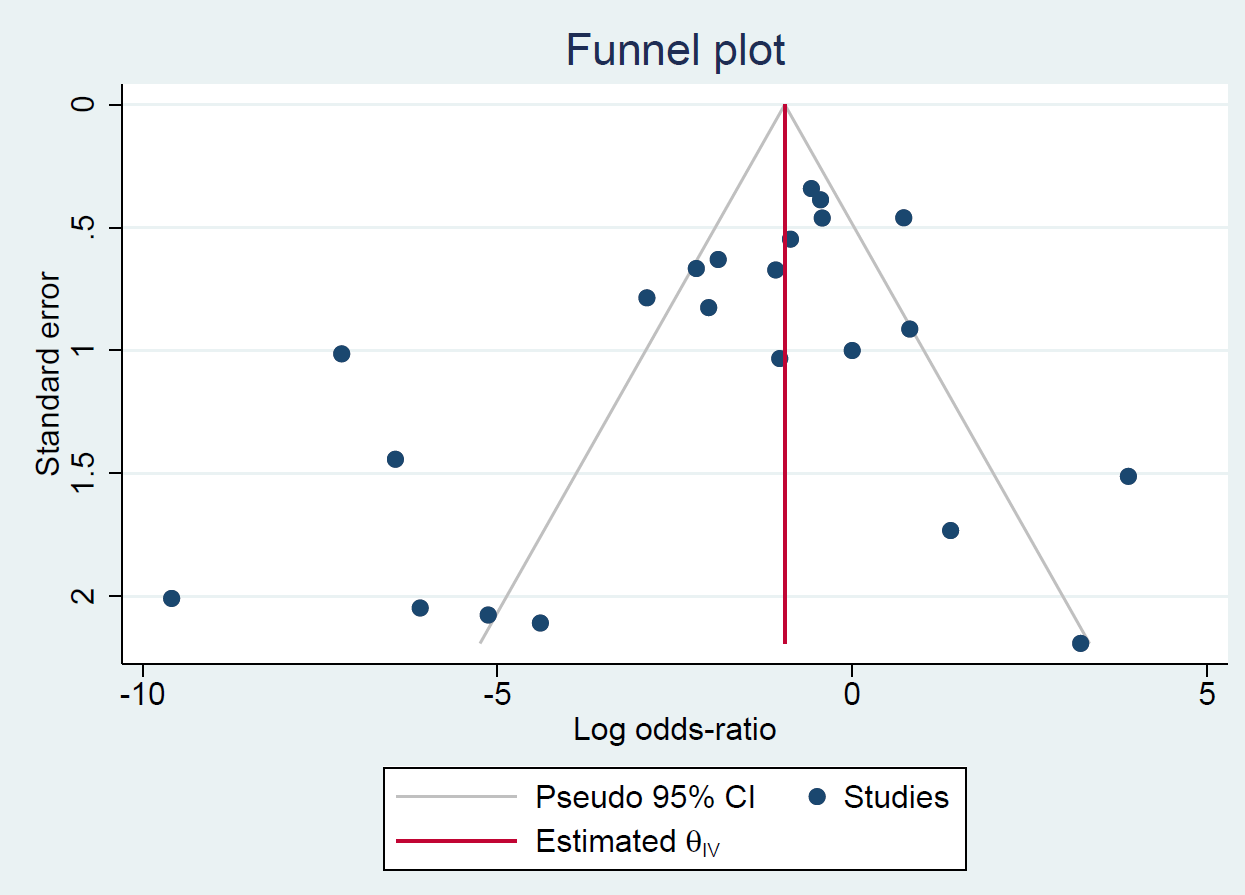


**Supplemental Figure S2.** Funnel plot indicating some extent of visual asymmetry for the diagnostic versus interventional procedure outcome.


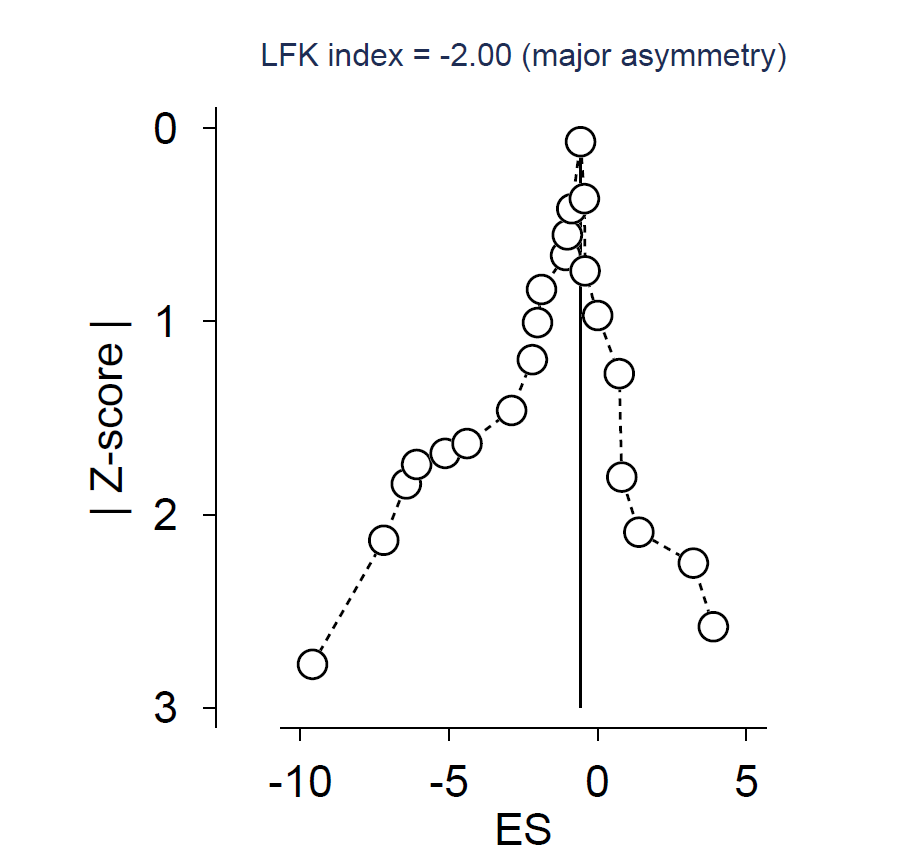


**Supplemental Figure S3.** Doi plot demonstrating a Luis Furuya-Kanamori (LFK) index of -2, suggestive of publication bias.
